# Retraction Note: Exogenous melatonin alleviates neuropathic pain-induced affective disorders by suppressing NF-κB/ NLRP3 pathway and apoptosis

**DOI:** 10.1038/s41598-026-65407-6

**Published:** 2026-08-10

**Authors:** Tahmineh Mokhtari, Lu-Peng Yue, Li Hu

**Affiliations:** 1 CAS Key Laboratory of Mental Health, Institute of Psychology, Chinese Academy of Sciences, Beijing, 100101 China; 2 Department of Psychology, University of Chinese Academy of Sciences, Beijing, 100049 China

Retraction of: *Scientific Reports* 10.1038/s41598-023-28418-1, published online 06 February 2023

The Editors have retracted this Article.

After publication, concerns were raised regarding the images presented in Fig. 6A:

PFC Sham-VEH and CCI-MLT images appear to overlap;

CA3 and DG CCI-VEH images appear to overlap.

The Authors were unable to provide verifiable raw data upon request.

Further checks by the Editors also found unusual features in the original western blot images presented in Figs. S1 and S2. The Editors therefore no longer have confidence in the presented data.

Lu-Peng Yue & Li Hu agree with this retraction. Tahmineh Mokhtari has not agreed to the retraction.

